# Supplementary material for: WBA: Word Boundary Attention for Chinese Named Entity Recognition
Source: PLoS One. 2025 Dec 23;20(12):e0336942. doi: 10.1371/journal.pone.0336942 (PMC12725666; doi:10.1371/journal.pone.0336942)
Supplement: Attachment — Submitted filename: Response to Reviewers.docx [file pone.0336942.s001.docx]

# Response to Reviewers

Dear Editors and Reviewers:

Thank you for your letter and for the editors’ and reviewers’ comments concerning our manuscript entitled “WBA: Word Boundary Attention for Chinese Named Entity Recognition” (ID: PONE-D-25-18226). Those comments are all valuable and very helpful for revising and improving our paper, as well as the important guiding significance to our researches. We have studied comments carefully and have made correction which we hope meet with approval. Revised portion are marked in red in the ‘Revised Manuscript with Track Changes’ file. The main corrections in the paper and the responds to the reviewer’s comments are listed below this letter.

We look forward to receiving your new comments.

Best wishes,

Zhongguo Xu

School of Computer Science and Technology, Tongji University

Shanghai 201804, China.

email: Zhongguo Xu@tongji.edu.cn

2025.06.18

**Journal requirements:**

When submitting your revision, we need you to address these additional requirements.

1. Please ensure that your manuscript meets PLOS ONE's style requirements, including those for file naming. The PLOS ONE style templates can be found at <https://journals.plos.org/plosone/s/file?id=wjVg/PLOSOne_formatting_sample_main_body.pdf> and https://journals.plos.org/plosone/s/file?id=ba62/PLOSOne_formatting_sample_title_authors_affiliations.pdf

**Response：**We ensure that our manuscript meets PLOS ONE's style requirements, including those for file naming.

2. We noticed you have some minor occurrence of overlapping text with the following previous publication(s), which needs to be addressed:

NFLAT: Non-Flat-Lattice Transformer for Chinese Named Entity Recognition - https://export.arxiv.org/pdf/2205.05832

In your revision ensure you cite all your sources (including your own works), and quote or rephrase any duplicated text outside the methods section. Further consideration is dependent on these concerns being addressed.

**Implement:** Page 4:Method;Page 8:Experiments

**Response：**We address some minor occurrence of overlapping text with the previous publication(s) in the revised portion marked in red in the paper.

3. Thank you for stating the following in the Acknowledgments Section of your manuscript:

“This work is supported by the National Natural Science Foundation of China (No. 34572071145).”

We note that you have provided funding information that is not currently declared in your Funding Statement. However, funding information should not appear in the Acknowledgments section or other areas of your manuscript. We will only publish funding information present in the Funding Statement section of the online submission form.

Please remove any funding-related text from the manuscript and let us know how you would like to update your Funding Statement. Currently, your Funding Statement reads as follows:

“The author(s) received no specific funding for this work.”

Please include your amended statements within your cover letter; we will change the online submission form on your behalf.

**Response：**We remove any funding-related text from the manuscript. We would like to update our Funding Statement and include our amended statements ‘This work is supported by the National Natural Science Foundation of China (No. 72071145).’ within our cover letter. Please change the online submission form on our behalf.

4. In the online submission form, you indicated that [Dataset can be downloaded upon request at \url{https://github.com/OYE93/Chinese-NLP-Corpus/tree/master/NER/Weibo},\url{https://paperswithcode.com/dataset/msra-cn-ner}，\url{https://catalog.ldc.upenn.edu/LDC2011T03} and \url{https://github.com/jiesutd/LatticeLSTM}].

All PLOS journals now require all data underlying the findings described in their manuscript to be freely available to other researchers, either 1. In a public repository, 2. Within the manuscript itself, or 3. Uploaded as supplementary information.

This policy applies to all data except where public deposition would breach compliance with the protocol approved by your research ethics board. If your data cannot be made publicly available for ethical or legal reasons (e.g., public availability would compromise patient privacy), please explain your reasons on resubmission and your exemption request will be escalated for approval.

**Response：**We Indicate that all data underlying the findings described in our manuscript is freely available to other researchers.

5. Please include a new copy of Table 4 in your manuscript; the current table is difficult to read. Please follow the link for more information: <https://blogs.plos.org/plos/2019/06/looking-good-tips-for-creating-your-plos-figures-graphics/>

**Response：**We include a new copy of Table 4 in 'Manuscript' file; we include a new copy of Table 4 in the ‘Revised Manuscript with Track Changes’ file with red mark.

**Responds to the reviewer’s comments:**

**Reviewer #1:** After a thorough review of the manuscript "WBA: Word Boundary Attention for Chinese Named Entity Recognition", I regret that I cannot recommend it for publication in PLOS ONE in its current form. The paper has several major flaws that significantly undermine its scientific contribution and validity.

**Major Concerns:**

Limited Novelty

The proposed WBA method appears to be a marginal extension of existing approaches like FLAT and NFLAT. The authors claim their method innovatively incorporates word boundary information, but this has already been extensively explored in previous works such as Lattice LSTM and FLAT. The "word boundary attention" mechanism seems to be merely a rebranding of conventional attention mechanisms without substantial theoretical innovation.

**Implement:** Page 3:Related Work and Background;Page 7: Equation 6 and 7

**Response：**Lattice LSTM is not use Transformer. NFLAT streamlines FLAT’s approach by computing attention scores solely for 'character-word' interactions. WBA computes 'character-character' attention scores using relative position encoding in Equation 6 and 7, incorporating both orientation and distance awareness. We incorporate multi-level word structure into character-level attention by leveraging the hybrid structure of words and characters in Chinese.

Methodological Flaws

The foundation of this work is questionable. The authors rely heavily on word segmentation tools or existing tags to obtain word boundary information, which introduces systematic errors and biases into the model. The relative position encoding scheme (Equations 6-9) lacks theoretical justification and appears arbitrary. The authors fail to demonstrate why their particular formulation is superior to simpler alternatives.

**Implement:** Page 7: Wordformer Attention, Equation 6 and 7

**Response：**1.After using word segmentation tools or existing tags to obtain word boundary information, the method estimates word position probabilities while handling ambiguous word boundaries to reduce systematic errors and biases into the model. It begins by enumerating all possible word boundary candidates. Next, it applies character-lexicon attention, using word match positions to compute the probability of each character appearing at the beginning (B), middle (M), end (E), or as a single-character word (S). Finally, the framework integrates masking and class attention mechanisms to generate informative position features for characters, leveraging both character and lexicon contexts.

2.We correct the Equations 6-9 to 6-7 and give theoretical justification of d^ph^ in Equation 6 and d^pt^ in Equation 7. It is superior to simpler alternatives because of the similarity with two relative distances.

Experimental Deficiencies

The experimental validation is inadequate in several aspects:

The reported 2.51% improvement on Weibo dataset is not convincing given the dataset's small size and high variance. No statistical significance tests were provided.

The comparison with state-of-the-art pre-trained models is incomplete and selective.

The ablation studies are insufficient to prove the necessity of each component.

**Implement:** Page 9: Table 3; Page 12: Table 4

**Response：**1.We make statistical significance tests with p<0.05 on Weibo dataset for the dataset's small size and high variance.

2. We make statistical significance tests with p<0.05 on all datasets.
3. We add ZEN pre-trained models to address the incomplete and selective comparison.
4. We should add ‘w/o Wordformer Attention’ in the ablation studies to prove the necessity of each component. We remove Wordformer Attention from NFLAT+WBA as NFLAT+self attention. For better understand, we add this experiment with discussion.

Results Analysis Issues

The visualization of word boundary attention (Figure 4) is superficial and potentially misleading. The authors interpret the attention patterns without rigorous quantitative analysis. The claim that WBA "effectively suppresses noise" is not supported by concrete evidence.

**Implement:** Page 13: Analysis of Word Boundary Attention

**Response：**1.We add rigorous quantitative analysis of word boundary attention (Figure 4).

2. We support "effectively suppresses noise" by concrete evidence with rigorous quantitative analysis.

**#question 1:**

Why should word boundaries be more informative than other linguistic features for NER?

**Implement:** Page 14: Why Word Boundaries More Informative

**Response：**1.Because different word boundaries will make different word types. Named Entity Recognition (NER) is traditionally viewed as a task involving both entity boundary detection and entity type classification. The main linguistic features are boundaries and types. The first step is boundary detection and the second step is type classification. For example, in text ‘南京市长江大桥’, ‘市’ is labeled as ‘E-Location’ meaning the end of location entity type for word boundary ‘南京市 (Nanjing City)’ while labeled as ‘B-Person’ meaning the beginning of person entity type for word boundary ‘市长 (Major)’. So word boundaries be more informative with word types.

2.Because no word boundaries no sentence structure. Language is equal word and structure. From word boundaries, we can find the different levels of structure in sentences for syntactic and dependency analysis. It will help for NER with graph structure embedding. So word boundaries is more informative with sentence structure.

**#question 2:**

How does the method handle ambiguous word boundaries?

**Implement:** Page 7: Wordformer Attention, Line 151-158

**Response：**The method calculates the word position probability handling ambiguous word boundaries. At first, we list all possible word boundaries for candidates. Then we consider character-lexicon attention with word match position and calculate the probability of characters appearing at the beginning (B), end (E), middle (M), and single (S) of words. Finally, our framework incorporates mask and class attention mechanisms to generate informative position features for characters, based on both character and lexicon contexts.

**#question 3:**

What is the theoretical upper bound for improvement using word boundary information?

**Implement:** Page 14-15: How WBA Brings Improvement, Line 357-370

**Response：**The theoretical upper bound for improvement using word boundary information for NER depends on entity type errors, word boundary errors and word composition errors. The errors from NER are entity boundary errors and entity type errors. A entity is a word or consist of words, so entity boundary errors is from word boundary errors and word composition errors. If we use word boundary information, the entity boundary errors will decrease. So the theoretical upper bound using word boundary information is no word boundary errors. Set entity type errors as $e_{t}$, word boundary errors as $e_{b}$ and word composition errors as $e_{c}$, the theoretical upper bound using word boundary information is $\bar{e_{t}\oplus e_{c}}$, the theoretical lower bound using word boundary information is $\bar{e_{t}\bigoplus e_{c}\bigoplus e_{b}}$, the improvement is $\bar{e_{t}\oplus e_{c}}-\bar{e_{t}\bigoplus e_{c}\bigoplus e_{b}}$.

**#question 4:**

Limited Applicability the authors acknowledge the method's language-specific nature but underestimate this limitation. The dependency on Chinese language features makes the contribution extremely narrow, especially for a general-scope journal like PLOS ONE.

**Response：**Although we study word boundary in Chinese language, it is also important in other language like ‘New York’ in English. It will inspire scholars to do the similarity work from general-scope journals like PLOS ONE.

**The authors need to:**

1. Substantially strengthen the theoretical foundation

**Implement:** Page 7: Wordformer Attention, Equation 6 and 7

**Response：**We correct the Equations 6-9 to 6-7 and give theoretical justification of d^ph^ in Equation 6 and d^pt^ in Equation 7. It is superior to simpler alternatives because of the similarity with two relative distances. Relative distances substantially strengthen the theoretical foundation.

2. Provide rigorous mathematical proofs for the proposed mechanisms

**Implement:** Page 7: Wordformer Attention, Equation 6 and 7

**Response：**We correct the Equations 6-9 to 6-7 and give theoretical justification of d^ph^ in Equation 6 and d^pt^ in Equation 7. It is superior to simpler alternatives because of the similarity with two relative distances. Relative distances provide rigorous mathematical proofs for the proposed mechanisms.

3.Conduct comprehensive experiments with statistical validation

**Implement:** Page 9: Table 3

**Response：**We make statistical significance tests with p<0.05 on all datasets.

4.Demonstrate clear advantages over existing methods

**Implement:** Page 13: How WBA Brings Improvement, Line 377-380

**Response：**Our method explicitly models word boundary attention across the entire sentence to mitigate word boundary errors, rather than merely incorporating word lexicons. This approach provides a more accurate representation of sentence structure and better captures relationships between nearby characters.

5.Address the fundamental questions about the approach's validity

**Implement:** Page 9: Table 3;Page 12: Table 4&5

**Response：**1.We make statistical significance tests with p<0.05 on all datasets. We Conduct comprehensive experiments with statistical validation about the approach's validity.

2. We add experiment in the ablation studies to prove the necessity of each component about the approach's validity.
3. We add pre-trained models to address the incomplete and selective comparison about the approach's validity.

6.In its current form, the manuscript reads more like an incremental technical report rather than a scientific contribution worthy of publication in PLOS ONE.

**Response：**We revise the manuscript to make a scientific contribution worthy of publication in PLOS ONE. Although we study word boundary in Chinese language, it is also important in other language like ‘New York’ in English. It will inspire scholars to do the similarity work from general-scope journals like PLOS ONE.

**Reviewer #2:** 1.The robustness of the model can be further discussed, such as its performance under noisy data or low-resource conditions, to enhance the credibility of the conclusions.

**Implement:** Page 12: Table 5

**Response：**We use few shots of datasets for low-resource conditions to enhance the credibility of the conclusions. To simulate few-shot scenarios, we randomly sample a varying number of samples from training set of Weibo and Resume training set while ensuring all label types are covered. The sampling sizes N are set to 250, 500, and 1000. The detailed sampling information for each label type and their corresponding Chinese label words (after mapping) follows FE-CFNER.

2.Statistical tests (e.g., t-test or ANOVA) can be added to validate the significance of performance improvements, especially for smaller improvements (e.g., 0.12% F1 score improvement on MSRA).

**Implement:** Page 9: Table 3

**Response：**We make statistical significance tests with p<0.05 on all datasets.

3.Professional English proofreading should be done before the final revision to ensure the accuracy of grammar and terminology.

**Response：**We revise the manuscript to ensure the accuracy of grammar and terminology worthy of publication in PLOS ONE.

**Reviewer #3:**

Abstract

1. Please give full form for WBA and YJ

**Implement:** Page 1: Abstract

**Response：**We give full form ‘Word Boundary Attention’ for WBA in Abstract;

’YJ’ denotes the lexicon released by Zhang and Yang (2018) and is full form.

Introduction

2. Line 9 and Line 65 The reference sequence number is missing

**Implement:** Page 2:Line 9 and Line 66

**Response：**We add the reference sequence number in Introduction.

3. Please reorganize the Introduction, Related Work, and Background section. Make them structured. You can add subtitles under Introduction section, but it is not good to have them in parallel.

**Implement:** Page 3:Related Work and Background

**Response：**We add Related Work and Background subtitles under Introduction section.

4. Please include the following papers in your Introduction:

*Li, Y., Peng, X., Li, J., Peng, S., Pei, D., Tao, C., Xu, H. and Hong, N., 2023, June. Development of a natural language processing tool to extract acupuncture point location terms. In 2023 IEEE 11th International Conference on Healthcare Informatics (ICHI) (pp. 344-351). IEEE.

*Lu, Q., Li, R., Wen, A., Wang, J., Wang, L. and Liu, H., 2024. Large language models struggle in token-level clinical named entity recognition. arXiv preprint arXiv:2407.00731.

*Li, Y., Viswaroopan, D., He, W., Li, J., Zuo, X., Xu, H. and Tao, C., 2025. Improving entity recognition using ensembles of deep learning and fine-tuned large language models: A case study on adverse event extraction from VAERS and social media. Journal of Biomedical Informatics, p.104789.

*Rehana, H., Zheng, J., Yeh, L., Bansal, B., Çam, N.B., Jemiyo, C., McGregor, B., Özgür, A., He, Y. and Hur, J., 2025. Cancer Vaccine Adjuvant Name Recognition from Biomedical Literature using Large Language Models. arXiv preprint arXiv:2502.09659.

*Li, Y., Li, J., He, J. and Tao, C., 2024. AE-GPT: using large language models to extract adverse events from surveillance reports-a use case with influenza vaccine adverse events. Plos one, 19(3), p.e0300919.

*Rehana, H., Bansal, B., Çam, N.B., Zheng, J., He, Y., Özgür, A. and Hur, J., 2024. Nested named entity recognition using multilayer BERT-based model. CLEF Working Notes.

*Li, Y., Peng, X., Li, J., Zuo, X., Peng, S., Pei, D., Tao, C., Xu, H. and Hong, N., 2024. Relation extraction using large language models: a case study on acupuncture point locations. Journal of the American Medical Informatics Association, 31(11), pp.2622-2631.

*He, J., Li, F., Li, J., Hu, X., Nian, Y., Xiang, Y., Wang, J., Wei, Q., Li, Y., Xu, H. and Tao, C., 2024. Prompt tuning in biomedical relation extraction. Journal of Healthcare Informatics Research, 8(2), pp.206-224.

*Li, Y., Viswaroopan, D., He, W., Li, J., Zuo, X., Xu, H. and Tao, C., 2025. Enhancing Relation Extraction for COVID-19 Vaccine Shot-Adverse Event Associations with Large Language Models. Research Square, pp.rs-3.

*Li, X., Zheng, Y., Hu, J., Zheng, J., Wang, Z. and He, Y., 2024. VaxLLM: Leveraging Fine-tuned Large Language Model for automated annotation of Brucella Vaccines. bioRxiv, pp.2024-11.

**Implement:** Page 1-2: Introduction

**Response：**We reference these papers in our Introduction.

Method

5. Where is Method section?

**Implement:** Page 4: Methods

**Response：**We replace the Model Architecture section as Method section.

6. Line 110, please revise: In this work, we implement this layer with different models different models,

**Implement:** Page 6: Line 112

**Response：**We delete the repetition of ‘different models’.

7. Line 112-114 not very clear: For base model, we use unigram and bigram embeddings in static character and word embedding layer. Our character embedding encoder embeds sentence on the character sequence c1, c2, . . . , cn and word sequence w1, w2, . . . , wm.

**Implement:** Page 6: Line 114-118

**Response：**We refine this contents to make clear our model. For base model, we use unigram and bigram embeddings in static character embedding layer. Each character ct is embedded as xct . Each word wt is embedded as xwt . The character embedding encoder represents the sentence based on the character sequence c1, c2, . . . , cn and the word embedding encoder represents the sentence based on word sequence w1, w2, . . . , wm.

8. Please explicitly explain Equation 3 and 4. The representations of some symbols like W_q_ were not given.

**Implement:** Page 6: Line 124-128;Page7: Line 149-150

**Response：**We give the representations of some symbols like W_q_, W_k_ and W_v_. Each of the projection matrices Wq , Wk, and Wv is a learnable parameter.

9. Line 154. where are dph and dpt in the Equations?

**Implement:** Page 7: Equation 6 and 7

**Response：**We correct the Equations and dph is in Equation 6 and dpt is in Equation 7.

10. This study have lots of findings in Ablation Study, Analysis of Word Boundary Attention, How WBA Brings Improvement, and How WBA Brings Improvement. I suggest you include your significant findings in Discussion section, and discuss the reasons.

**Implement:** Page 10: Discussion

**Response：**We include our significant findings in Discussion section, and discuss the reasons.
