# Supplementary material for: WBA: Word Boundary Attention for Chinese Named Entity Recognition
Source: PLoS One. 2025 Dec 23;20(12):e0336942. doi: 10.1371/journal.pone.0336942 (PMC12725666; doi:10.1371/journal.pone.0336942)
Supplement: Attachment — Submitted filename: Response to Reviewers-9.5.docx [file pone.0336942.s002.docx]

# Response to Reviewers

Dear Editors and Reviewers:

Thank you for your letter and for the editors’ and reviewers’ comments concerning our manuscript entitled “WBA: Word Boundary Attention for Chinese Named Entity Recognition” (ID: PONE-D-25-18226R1). Those comments are all valuable and very helpful for revising and improving our paper, as well as the important guiding significance to our researches. We have studied comments carefully and have made correction which we hope meet with approval. Revised portion are marked in red in the ‘Revised Manuscript with Track Changes’ file. The main corrections in the paper and the responds to the reviewer’s comments are listed below this letter.

We look forward to receiving your new comments.

Best wishes,

Zhongguo Xu

School of Computer Science and Technology, Tongji University

Shanghai 201804, China.

email: Zhongguo Xu@tongji.edu.cn

2025.09.15

**Journal requirements:**

If the reviewer comments include a recommendation to cite specific previously published works, please review and evaluate these publications to determine whether they are relevant and should be cited. There is no requirement to cite these works unless the editor has indicated otherwise.

**Response:** This round of reviewer comments did not request additional citations. In the previous review, some specific references were suggested, and we conducted a thorough review and evaluation before including them.

**Responds to the reviewer’s comments:**

### ****Response to Reviewer #1****

**Comment 1 (Language and Style):**

Language and Style: A final pass for grammar and fluency is recommended. While much improved, occasional awkward phrasing remains (e.g., “no word boundaries no sentence structure” could be rephrased for clarity).
**Summary：**You suggested a final pass for grammar and fluency, e.g., rephrasing “no word boundaries no sentence structure.”

**Revision Location：** Page 17: Why Word Boundaries More Informative , Previous response to reviewers.

**Response:**

We have thoroughly proofread the manuscript and improved awkward phrasing. The sentence has been revised to: “Without word boundaries, sentence structure cannot be effectively represented.”

**Comment 2 (Limitation Framing):**

Limitation Framing: The limitation section is appropriate, but consider softening the claim that the method “may not be effective for English.” Instead, note that it is optimized for Chinese and that future work could explore cross-lingual adaptation.
**Summary：**You recommended softening the claim about ineffectiveness for English.

**Revision Location：**Page 18: Limitations

**Response:**
We have revised the limitations section to: “Future work could explore cross-lingual adaptation. It could potentially work for ...”

**Comment 3 (Figure Readability):**

Figure Readability: Ensure that Figure 4 (now revised) is legible in print and grayscale formats, as per PLOS guidelines.
**Summary：**Ensure Figure 4 is legible in print and grayscale.

**Revision Location：**Page 12: Figure 4

**Response:**
We have adjusted Figure 4 by enhancing contrast .The figure is now tested for readability in grayscale, as shown below. We label colors in caption of Figure 4 .

### ****Response to Reviewer #2****

**Comment 1 (Clarify Novelty):**

Clarify the Novelty – More explicitly highlight how WBA differs from FLAT and NFLAT beyond rebranding. A clearer articulation of the theoretical innovation would strengthen the contribution.
**Summary：**You asked for a clearer articulation of how WBA differs from FLAT and NFLAT.

**Revision Location：**Page 4:Related Work and Background;Page 8: Equation 6 and 7

**Response:**

We have added content in Background explicitly comparing WBA with FLAT and NFLAT, highlighting that WBA introduces a novel **word boundary guided relative position encoding**, rather than lattice-based fusion. NFLAT streamlines FLAT’s approach by computing attention scores solely for 'character-word' interactions. WBA computes 'character-character' attention scores using relative position encoding in Equation 6 and 7, incorporating both orientation and distance awareness. We incorporate multi-level word structure into character-level attention by leveraging the hybrid structure of words and characters in Chinese.

**Comment 2 (Enhance Visualization and Analysis):**

Enhance Visualization and Analysis – The current visualizations are helpful but somewhat superficial. Adding quantitative measures (e.g., entropy of attention distributions, cross-dataset consistency) would provide stronger evidence for the claimed noise suppression.
**Summary：**You suggested adding quantitative measures for attention interpretability.

**Revision Location：**Page 14-15:Analysis of Word Boundary Attention,Fig.5-7

**Response:**
We have included **attention entropy analysis** and **cross-dataset consistency tests** (see Fig.5-7) to quantitatively support the claim of noise suppression.

**Comment 3 (Improve Theoretical Clarity):**

Improve Theoretical Clarity – The explanations around Equations (6)–(11) remain verbose. Presenting the meaning of key variables (e.g., dph, dpt) in a more intuitive form, such as through diagrams or simplified examples, would improve readability.
**Summary：**Equations (6)–(11) are verbose; suggested diagrams or simplified examples.

**Revision Location：**Page 7：Wordformer Attention

**Response:**
We have referred in an illustrative diagram (Figure 3) and a toy example to explain dph and dpt more intuitively.

**Comment 4 (Extend Experimental Coverage):**

Extend Experimental Coverage – While the focus is on Chinese NER, including at least one experiment on a non-Chinese dataset (e.g., CoNLL-2003 in English) would demonstrate broader applicability and address concerns about language specificity.
**Summary：**You recommended testing on a non-Chinese dataset.

**Response:**
We regret that our method is not suitable for experiments on the CoNLL-2003 English dataset. Our approach relies on word boundary attention, where head and tail nodes are defined for words in Chinese. However, in English, head and tail nodes are nearly identical, as word boundaries are explicit. Thus, word boundary attention in English effectively reduces to standard self-attention.

**Comment 5 (Polish Language and Style):**

Polish Language and Style – Several grammatical and stylistic issues remain (e.g., awkward phrasing in the abstract). A round of professional English editing would improve clarity and make the paper more accessible to an international audience.
**Summary：**You recommended professional editing.

**Response:**
We have thoroughly polished the language and also used an English editing service for additional fluency improvements.

### ****Response to Reviewer #3****

**Comment 1 (Figure Caption Consistency):**
**Summary：**You noted inconsistency between “Fig 1” and “Figure 1a/b.”

**Response:**
We have unified all figure references to “Fig. X” format.

**Comment 2 (Reference Formatting):**
**Summary：**Reference 26 and 27 author formats incorrect.

**Response:**
We have corrected the references according to the journal’s required format.

### ****Response to Reviewer #4****

**Comment 1 (Clarify Difference from Transformer):**

The proposed Word Boundary Attention (WBA) employs Transformer attention technique to solve the NER task. The authors should clarify how this method differs from the standard Transformer architecture and highlight the specific innovations.
**Summary：**You suggested clarifying how WBA differs from the standard Transformer.

**Response:**
We have added explanation in Page 7：Wordformer Attention, noting that WBA modifies the attention mechanism by integrating **word boundary relative position encoding**, which is absent in vanilla Transformers. Unlike the standard Transformer, which applies self-attention uniformly across tokens, WBA introduces explicit head and tail nodes to represent word boundaries. This modification allows the model to capture word-level structural information, which is critical for Chinese where word segmentation is ambiguous. Furthermore, WBA restricts and reweights attention based on boundary positions, thereby reducing noise from irrelevant characters. These innovations distinguish WBA from standard attention and contribute directly to improved NER performance.

**Comment 2 (Expand Related Work):**

The authors could enrich the manuscript by providing a more extensive review of related work.
**Summary：**You recommended enriching the related work review.

**Response:**
We have expanded related work(Page 3-4) with a more comprehensive survey of recent Chinese NER models (including ZEN, BERT-wwm, PLMs) and structural encoding methods.

**Comment 3 (Computational Cost and Scalability):**

Although the WBA shows some improvements, a deeper analysis of the computational cost and scalability would be beneficial to fully understand its practical applications.
**Summary：**You asked for deeper analysis of computational aspects.

**Response:**
We have added a Complexity Analysis in page 11 comparing mode size and time cost among WBA, TENER, NFLAT and FLAT. Importantly, NFLAT(YJ)+WBA maintains a computational cost comparable to NFLAT(YJ) while remaining significantly more efficient than FLAT(YJ).

**Comment 4 (Ablation Study):**

The ablation study is not sufficiently comprehensive. It is recommended to include more thorough ablation experiments.
**Summary：**You suggested more comprehensive ablations.

**Response:**
We have extended the ablation study (see Table 5,page 13) to include experiments isolating **relative position encoding**, **boundary guidance**, and **lexicon integration**, which confirm the effectiveness of each component. Taken together, the ablation experiments demonstrate that Wordformer is the most influential component in our framework. Even when lexicon fusion or the Transformer encoder are removed, Wordformer alone yields higher performance than strong baselines, particularly on the low-resource Weibo dataset. This shows that explicit boundary modeling provides substantial benefits beyond existing mechanisms, validating Wordformer as the central source of improvement in our method.
