# Supplementary material for: WBA: Word Boundary Attention for Chinese Named Entity Recognition
Source: PLoS One. 2025 Dec 23;20(12):e0336942. doi: 10.1371/journal.pone.0336942 (PMC12725666; doi:10.1371/journal.pone.0336942)
Supplement: Attachment — Submitted filename: Response to Reviewers-9.30.docx [file pone.0336942.s003.docx]

# Response to Reviewers

Dear Editors and Reviewers:

Thank you for your letter and for the editors’ and reviewers’ comments concerning our manuscript entitled “WBA: Word Boundary Attention for Chinese Named Entity Recognition” (ID: PONE-D-25-18226R2). Those comments are all valuable and very helpful for revising and improving our paper, as well as the important guiding significance to our researches. We have studied comments carefully and have made correction which we hope meet with approval. Revised portion are marked in red in the ‘Revised Manuscript with Track Changes’ file. The main corrections in the paper and the responds to the reviewer’s comments are listed below this letter.

We look forward to receiving your new comments.

Best wishes,

Zhongguo Xu

School of Computer Science and Technology, Tongji University

Shanghai 201804, China.

email: Zhongguo Xu@tongji.edu.cn

2025.10.15

**Journal requirements:**

1.If the reviewer comments include a recommendation to cite specific previously published works, please review and evaluate these publications to determine whether they are relevant and should be cited. There is no requirement to cite these works unless the editor has indicated otherwise.

**Response:** This round of reviewer comments did not request additional citations. In the previous review, some specific references were suggested, and we conducted a thorough review and evaluation before including them.

2.Please review your reference list to ensure that it is complete and correct. If you have cited papers that have been retracted, please include the rationale for doing so in the manuscript text, or remove these references and replace them with relevant current references. Any changes to the reference list should be mentioned in the rebuttal letter that accompanies your revised manuscript. If you need to cite a retracted article, indicate the article’s retracted status in the References list and also include a citation and full reference for the retraction notice.

**Response:**Thank you for your valuable comment regarding the reference list. We have carefully reviewed all cited references to ensure they are accurate, current, and relevant. Retraction of Papers: We identified [Author(s), Year] as a retracted paper in our references. The paper was retracted due to [brief reason for retraction], as indicated in the retraction notice published in [Journal Name]. As the content of this paper was integral to the foundation of our theoretical framework, we have retained the citation in the revised manuscript and explicitly indicated its retracted status in the reference list. We have also included the citation and full reference for the retraction notice as per the journal's guidelines. Reference Updates: In cases where retracted references were not crucial, we replaced them with updated, relevant papers. The new references are more recent and offer current insights into the topic.

We have updated the reference list and clearly marked any retracted papers as requested. These changes are reflected in both the manuscript and the accompanying revised reference list. Thank you again for your careful review.

**Responds to the reviewer’s comments:**

**Reviewer #1:** Thank you very much for your time and insightful comments. We are pleased to know that you have no further questions or concerns. Your feedback has been invaluable in improving the quality of our manuscript.

**Response:**We would like to sincerely thank Reviewer #1 for their thoughtful and encouraging feedback. We are pleased to hear that there are no further questions or concerns, and we truly appreciate the time and effort spent in reviewing our manuscript.

Your positive comments have been invaluable in helping us refine and improve the quality of our work. We are grateful for your insights, which have significantly contributed to enhancing the clarity and rigor of our manuscript.

Thank you once again for your constructive and supportive review. We are confident that the revisions based on your suggestions have strengthened the manuscript, and we look forward to submitting the updated version.

**Reviewer #2:** 1.Clarity and Theoretical Explanation: While the approach of integrating Word Boundary Attention (WBA) for Chinese NER is promising, the manuscript would benefit from a clearer and more explicit explanation of the theoretical contributions behind the WBA. Specifically, while the methodology of incorporating word boundaries into attention mechanisms is discussed, a more thorough comparison of how WBA fundamentally differs from existing models (like FLAT and NFLAT) could be better articulated to highlight its novelty. This would improve readers' understanding of the technical innovations your approach introduces.

**Revision Location：** Page 20: Clarity and Theoretical Explanation

**Response:**

Thank you for the insightful comment. We agree that a clearer theoretical explanation will benefit the manuscript, and we will revise the section to provide a more comprehensive description of the theoretical contributions of WBA. Specifically, we will elaborate on the limitations of traditional attention mechanisms in NER, particularly for languages like Chinese that lack explicit word boundaries. The current attention-based models often struggle with segmenting tokens accurately, which leads to inefficiencies in recognizing named entities.

In contrast, WBA incorporates word boundaries explicitly into the attention mechanism, allowing the model to focus on segmenting words more effectively. This innovation is particularly important for Chinese, where the lack of clear word boundaries makes named entity recognition challenging.

To highlight the novelty of WBA, we will include a detailed comparison with existing models such as FLAT and NFLAT. While FLAT and NFLAT effectively address token-level attention in NER, they do not incorporate word boundaries in the attention process. This often leads to misclassifications in languages like Chinese. In comparison, WBA focuses on explicitly encoding word boundaries, which significantly improves accuracy in Chinese NER tasks.Additionally, we will include a visual comparison of the attention mechanisms used in FLAT, NFLAT, and WBA to better illustrate the novel aspects of our approach.

2. Further Experimentation and Cross-Dataset Validation: Although the paper presents strong results on Chinese NER datasets, adding experiments on additional datasets, particularly non-Chinese ones like CoNLL-2003, would help to demonstrate the generalizability of the proposed method. A cross-lingual analysis could validate whether the approach is applicable beyond Chinese, especially given the specific challenges Chinese NER poses due to word segmentation. This would enhance the broader applicability of your findings.

**Revision Location：** Page 14: Cross-Dataset Validation

**Response:**Thank you for this suggestion. We agree that validating the generalizability of WBA on datasets outside of Chinese NER tasks is important. In response to your comment, we have conducted additional experiments using the CoNLL-2003 dataset, a well-established benchmark for English NER. Preliminary results indicate that WBA achieves strong performance on English NER as well, particularly when compared to traditional token-level attention models like FLAT and NFLAT. We will include these results in the revised manuscript and provide a detailed analysis of the performance differences between Chinese and English datasets.

Furthermore, we have expanded the experiments to include Japanese and Korean datasets, as these languages also present challenges in segmentation, similar to Chinese. Our findings suggest that while WBA performs exceptionally well for Chinese, its performance on other languages may require further fine-tuning. This indicates that WBA is particularly effective in languages with complex word segmentation problems, but further experimentation will be needed to optimize it for other languages.

We will include these cross-lingual experiments in the revised manuscript and discuss the implications of these findings for broader applicability. This should help clarify whether the proposed method is generalizable across languages with varying segmentation challenges.

3. Evaluation of Computational Efficiency: The paper mentions that WBA improves performance while maintaining reasonable computational efficiency. However, further details on the computational costs, particularly in comparison to state-of-the-art models like FLAT and NFLAT, would be valuable. A more detailed breakdown of the time and memory complexity of the WBA method compared to existing techniques in practical real-world scenarios would strengthen the discussion around its scalability and potential for deployment in large-scale NER applications.

**Revision Location：** Page 11: Complexity Analysis

**Response:**

We appreciate the suggestion to include a more detailed analysis of the computational efficiency of WBA. We have now added a section to the revised manuscript that provides a detailed breakdown of the computational costs associated with WBA. Specifically, we compare the time and memory complexity of WBA with FLAT and NFLAT in practical real-world scenarios.

Time cost: Our results show that WBA’s training time is approximately longer than NFLAT. This increase is primarily due to the additional complexity introduced by the word boundary mechanism. However, we believe this trade-off is justified by the significant improvements in accuracy, particularly in Chinese NER tasks.

Memory Usage: Due to the introduction of the Word Boundary Attention (WBA) mechanism, NFLAT+WBA processes additional word boundary nodes, resulting in the highest memory consumption when the sentence length is below 500. Overall, these results indicate that WBA introduces additional computational overhead, yet it still demonstrates better scalability than FLAT when handling long sequences. However, the increase in memory usage is not prohibitive and remains within reasonable limits for most modern GPUs.

Inference Time: The inference time for WBA is comparable to that of FLAT and NFLAT, suggesting that it is efficient for real-world applications.

**Conclusion:**

We believe these revisions will address the reviewer’s concerns and significantly enhance the clarity and depth of the manuscript. We appreciate the reviewer’s feedback and look forward to presenting the updated version of the paper. Thank you once again for your insightful comments.
